# Machine learning approach for quantitative biodosimetry of partial-body or total-body radiation exposures by combining radiation-responsive biomarkers

**DOI:** 10.1038/s41598-023-28130-0

**Published:** 2023-01-18

**Authors:** Igor Shuryak, Leah Nemzow, Bezalel A. Bacon, Maria Taveras, Xuefeng Wu, Naresh Deoli, Brian Ponnaiya, Guy Garty, David J. Brenner, Helen C. Turner

**Affiliations:** 1 Center for Radiological Research, Columbia University Irving Medical Center, 630 West 168th street, VC-11-234/5, New York, NY 10032 USA; 2 Radiological Research Accelerator Facility, Columbia University Irving Medical Center, Irvington, NY USA

**Keywords:** Biological models, Experimental organisms, Proteomic analysis, Software, Computational biology and bioinformatics, Biomarkers, Risk factors

## Abstract

During a large-scale radiological event such as an improvised nuclear device detonation, many survivors will be shielded from radiation by environmental objects, and experience only partial-body irradiation (PBI), which has different consequences, compared with total-body irradiation (TBI). In this study, we tested the hypothesis that applying machine learning to a combination of radiation-responsive biomarkers (ACTN1, DDB2, FDXR) and B and T cell counts will quantify and distinguish between PBI and TBI exposures. Adult C57BL/6 mice of both sexes were exposed to 0, 2.0–2.5 or 5.0 Gy of half-body PBI or TBI. The random forest (RF) algorithm trained on ½ of the data reconstructed the radiation dose on the remaining testing portion of the data with mean absolute error of 0.749 Gy and reconstructed the product of dose and exposure status (defined as 1.0 × Dose for TBI and 0.5 × Dose for PBI) with MAE of 0.472 Gy. Among irradiated samples, PBI could be distinguished from TBI: ROC curve AUC = 0.944 (95% CI: 0.844–1.0). Mouse sex did not significantly affect dose reconstruction. These results support the hypothesis that combinations of protein biomarkers and blood cell counts can complement existing methods for biodosimetry of PBI and TBI exposures.

## Introduction

A large-scale radiological event such as improvised nuclear device detonation during a conflict between countries or terrorist activities can expose vast numbers of people to ionizing radiation. Since physical dosimeters are not available to the general population, reconstruction of radiation doses based on easily accessible biofluids (*e.g.* blood) from exposed individuals is important for making appropriate treatment decisions, and for providing information to the affected persons^1,2^. This forms the rationale for the field of radiation *biodosimetry*.

Due to partial shielding of radiation by objects like vehicles, building materials or equipment, many exposed survivors will be irradiated non-homogeneously. This situation applies mainly to the extremely high dose rate “prompt” exposures which occur within the first fraction of a second after a nuclear device detonation^3^. Such partial body irradiation (PBI) has important consequences for medical treatment and radiation-induced disease progression^4–6^. For example, hematopoietic system recovery after high-dose irradiation is facilitated by shielding a portion of the bone marrow (*e.g.* one or more limbs)^6–8^. Animal experiments show that even 5% bone marrow shielding improves survival from hematopoietic acute radiation syndrome (H-ARS)^9^ and affects the gastrointestinal (GI) syndrome. Erroneous misclassification of partial body exposure as total body exposure to a lower dose could overestimate the risk of H-ARS and underestimate late complication risks (*e.g.,* carcinogenesis) in the irradiated organs.

Consequently, it is important to search for reliable and high-throughput biodosimetry methods for PBI as well as for TBI scenarios^10–20^. Published literature suggests that blood cell counts and protein biomarkers provide promising opportunities for detecting and quantifying partial body exposures in animals^20,21^ and in human cancer patients treated with radiotherapy^13,14^. For example, heterogeneous exposures could be distinguished from homogeneous ones 24 h post irradiation by elevated dispersion of γ-H2AX foci^14^. In our group at the Center for Radiological Research (CRR) at Columbia University, we have developed the FAST-DOSE (Fluorescent Automated Screening Tool for Dosimetry) assay device, based on novel imaging flow cytometry (IFC)^22^. It uses a panel of radio-responsive intracellular biomarkers to rapidly quantify the upregulation of biomarker expression in blood leukocytes using fluorescent imaging and algorithms for the estimation of absorbed dose. The biomarkers have shown robust and persistent radiation dose responses after acute radiation exposure of humanized mice^22,23^. The advantage of the FAST-DOSE biomarker platform is that it provides rapid time to result by utilizing high throughput sample preparation, image capture and analysis^22–24^.

Previous studies have made notable progress for the detection and quantification of PBI exposures using biodosimetry assays including γ-H2AX, chromosome aberrations, gene expression, as well as several promising methods developed for automated, high-throughput scoring of cytogenetic endpoints^10,13–15,17,25–31^. Large-scale studies such as inter-laboratory comparisons reveal that the accuracy of biodosimetry for PBI scenarios still remains limited^29^, and PBI detection methods need to be tested and validated against TBI. Therefore, there is a need for development of new methods to complement existing (*e.g.,* cytogenetic) techniques^32,33^.

In this study, we investigated the hypothesis that state of the art ensemble machine learning (ML) methods such as random forest (RF) can be useful for combining the dose responses of radiation-responsive biomarkers (ACTN1, DDB2, FDXR) and blood cell counts (B and T cells) to perform quantitative biodosimetry under PBI or TBI conditions. We used male and female C57BL/6 mice, exposed to TBI or half-body PBI, as the model system for this investigation.

The selected biomarkers are known to be involved in mechanisms of radiation-induced damage response and/or repair, and cellular homeostasis^22,23^. Ferredoxin reductase (FDXR) is a mitochondrial flavoprotein that initiates electron transport for cytochromes P450 receiving electrons from NADPH^34^. Damage specific DNA binding protein 2 (DDB2) is the smaller subunit of a heterodimeric protein complex that participates in nucleotide excision repair, and this complex mediates the ubiquitylation of histones H3 and H4, which facilitates the cellular response to DNA damage^35^. Actinin Alpha 1 (ACTN1) is a cytoskeletal protein with multiple roles, including radiation responses, in different cell types^36^.

We present data compiled from TBI and PBI exposed mice, and sham-exposed controls. The total exposure dose (G_y_) was represented by the Dose variable. The distinction between TBI and PBI was represented by the Interaction variable, which was set to 1.0 × Dose for TBI and 0.5 × Dose for PBI. The Dose and Interaction variables were treated as outcome (target) variables which had to be predicted by ML as biodosimetry tasks. We also used ML to classify irradiated samples into PBI or TBI categories. The main predictor variables in these analyses were log-transformed B and T blood cell counts, normalized fluorescence values for the top-performing biomarkers, and cell surface markers (percentages of B and T cells among all cells). We also considered mouse sex and radiation type (electrons or x rays) as additional potential predictors, which could influence the results. We believe that this study provides a useful contribution to the field of biodosimetry of partial-body exposures by integrating hematological parameters with radiation-responsive protein biomarkers in an ML framework.

## Materials and methods

### Experimental procedures

The mouse experiments were approved by the Columbia University Institutional Animal Care and Use Committee (IACUC, approved protocol #AABA9506) and were conducted under all relevant federal and state guidelines. Male and female C57BL/6 mice aged (aged 12–14 weeks) were purchased from Charles River Laboratories (Frederick, MD) and randomly assigned to the sham (0 Gy) and irradiated (2.0–2.5 and 5 Gy) study groups. A summary of the numbers of mice in each exposure group is provided in Table 1, and the full data are provided in Supplementary_table_S1 online. All methods were performed in accordance with ARRIVE guidelines (https://arriveguidelines.org) and with other relevant guidelines and regulations.

**Table 1:** Summary of the number of mice in each exposed group.

Experiment	Date of the experiment	Radiation type	Assigned doses (Gy)	Number of mice
PBI, males, batch 1	7/14/2021	Electrons	0.0	4
			2.5	3
			5.0	4
PBI, males, batch 2	9/08/2021	Electrons	0.0	3
			2.5	3
			5.0	4
PBI, females, batch 3	02/01/2022	Electrons	0.0	5
			2.5	3
			5.0	3
PBI, females, batch 4	06/09/2022	Electrons	0.0	4
			2.5	3
			5.0	3
TBI, males, batch 1	7/14/2021	Electrons	0.0	3
			2.5	3
			5.0	3
TBI, males, batch 2	9/08/2021	Electrons	0.0	3
			2.5	4
			5.0	3
TBI, females, batch 3	02/01/2022	Electrons	0.0	3
			2.5	1
			5.0	3
TBI, females, batch 4	06/09/2022	Electrons	0.0	4
			2.5	3
			5.0	3
TBI, males	03/23/2022	X-rays	0.0	5
			2.0	5
			5.0	5
TBI, females	02/01/2022	X-rays	0.0	4
			2.0	3
			5.0	3

As described in the main text, detailed dosimetry was performed on each mouse and dose variations around the nominally assigned values were accounted for in the analysis, and two mouse samples in the TBI females batch 3 group were excluded from analysis due to insufficient number of B and T cells for scoring. In total, 42 mice were assigned to PBI (16 of them sham-exposed) and 36 mice to TBI exposures (13 of them sham-exposed) using CLINAC electrons, and 25 mice were assigned to x-ray exposures (9 of them sham-exposed).

### Irradiation and dosimetry

#### Clinac

PBI and TBI exposures were performed at the Radiological Research Accelerator Facility (RARAF), using 9 MeV electrons generated by our modified Clinac 2100C^37^. Batches of mice were irradiated on different dates, with random assignment of the mice to exposure type and dose. Mice were anesthetized using isofluorane and placed into a custom irradiation jig with a movable ¼ inch thick lead shield of the lower half of the body (for PBI exposures), or no shielding for TBI. The jig was placed at a source to surface distance of 90 cm and dose was delivered at a dose rate of 5–10 Gy/sec, which ensured that the circulation time of blood in the mouse, ~ 15 s^38^, was much longer than the dose delivery time (≤ 1 s).

Dose rate was evaluated prior to the experiment using a NIST-traceable advanced Markus ion chamber and Unidos E electrometer (PTW, Germany). The jig was placed at a source to surface distance of 90 cm and dose was delivered at a dose rate of 7 Gy/sec (~ 0.4 Gy/pulse @ 180 Hz). The number of Clinac pulses required to deliver 2.5 or 5 Gy was evaluated prior to the experiment using a NIST-traceable advanced Markus ion chamber and Unidos E electrometer (PTW, Germany). 2.5 Gy irradiations required 65 pulses and 5 Gy irradiations required 130 pulses, each after 20 s warm up time in which the electron gun was active but no dose was delivered^37^. To verify dose on a per-mouse basis, EBT3 film (Ashland, Bridgewater, NJ) was irradiated with each mouse. The film was scanned using an V700 photo scanner (Epson, Suwa Japan)^39^ and dose was reconstructed from the red channel data using the previously determined calibration curve:$D\left[Gy\right]=\frac{7.404 OD}{0.818-OD}$, where the optical density, *OD,* is the negative log transformed ratio of the pixel values (red channel only) of exposed and unexposed film, scanned simultaneously. Dose variation through the mouse thickness was previously measured to be about 10% in this irradiation geometry.

The experimental plan was to irradiate 4 batches of mice, where each batch included irradiated mice and corresponding controls which were sham-irradiated with the corresponding TBI or PBI procedures. The samples from 2 female mice exposed to 2.5 Gy TBI were excluded from analysis due to very low levels of B and T cells, insufficient for scoring (Table 1). Consequently, the analyzed data set (Supplementary_table_S1 online) included 42 animals exposed to Clinac PBI and 36 animals exposed to TBI.

#### X-RAD

For comparison, 25 mice (15 male; 10 female) were irradiated with 0, 2 or 5 Gy of TBI exposures using 320 kVp x-rays, a current of 12.5 mA, and dose rate of 1 Gy/min, using the X-RAD 320 biological irradiator (Precision X-Ray Inc, North Branford, CT) at the Center for Radiological Research. This additional data set enabled us to increase the sample size of the study, and to compare the effects of different types of radiations. Mouse irradiations were performed according to previous protocols^22,40^. For in-vivo irradiations, mice were placed in a specifically designed mouse irradiation holder (Precision X-ray). Control mice were sham irradiated. All doses were validated using a Radcal ion chamber (Monrovia, CA) placed in the mouse holder. During the actual irradiations, the delivered dose was measured by placing the ion chamber at the same position into the mouse holder. These x-ray exposures were performed to compare TBI exposures to high dose rate Clinac electrons at 5–10 Gy/sec with TBI exposures to lower dose rate x-rays at 1 Gy/min.

#### Blood sample collection and cell counts

All irradiated and sham-control mice were euthanized by CO_2_ asphyxiation at 24 h after radiation exposure to mimic realistic scenarios of biodosimetry measurements following a mass radiological event. Peripheral whole blood (WB) samples were collected from each mouse by cardiac puncture using a heparin-coated syringe prepared by adding 500 µl DPBS to BD Vacutainer containing 158 USP units of sodium heparin (#366,480). Similar to our earlier work^22^, leukocyte, T and B cell counts were determined by flow cytometry (CytoFLEX, Beckman Coulter, Pasedena, CA) using 20 μL of heparinized blood, using the following antibodies purchased from Biolegend (San Diego, CA): APC-CD45 (catalog #103,112), FITC-CD3e (#100,306), PE-CD19 (#115,508). Blood counts were determined using CytExpert software (Beckman Coulter).

#### Imaging flow cytometry (IFC) analysis

Peripheral WB samples (100 µl) from each mouse were aliquoted into matrix tubes (Thermo Scientific; #3740TS) for sample staining and fixing as follows: Erythrocytes in mock and x-irradiated mouse peripheral blood were lysed with RBC Lysis Buffer (eBioscience #00–4333-57), and remaining leukocytes were surface stained for 15 min, in the dark, at room temperature with anti-mouse CD3 PE (eBioscience; #12–0031-82) and anti-mouse CD19 PE/Dazzle (Biolegend; #115,554) T-cell and B-cell markers, respectively. Surface-stained leukocytes were washed in 1% BSA, then fixed and permeabilized for 20 min at 4 °C (BD Biosciences; Cytofix/Cytoperm; #554,714) and washed per manufacturer instructions, and intracellularly stained overnight, at 4 °C, with one of the following antibodies: FDXR (Sigma; #HPA044393), ACTN1 (Cell Signaling Technology; #3134 s). DDB2-FITC (Cusabio; #CSB-PA846067LC01HU) antibody incubation occurred in the dark for 1 h at room temperature. Except for DDB2 (which is a FITC conjugated antibody), all other samples stained with primary intracellular antibodies were then stained for 1 h, in the dark at room temperature, with goat anti-rabbit Alexa Fluor 488 secondary antibody (Life Technologies; #A11034). The antibody dilutions were: ACTN 1:100, DDB2 1:100, FDXR 1:100, CD3 1:800, CD19 1:800, AF488 goat anti-rabbit 1:1000. Cells were then washed with and stored in DPBS at 4 °C until scanning.

Single, focused cells (approximately 3000) per sample were acquired on the ImageStream MkII Imaging Flow Cytometer (Luminex, Austin, TX) with the 488 nm at 200 mW laser power at 40 × magnification. To compensate for spectral spillover, cells stained with single fluorescence only were acquired using the compensation wizard on INSPIRE software (488 nm laser on with the brightfield and side scatter inactivated). The compensation coefficients were determined automatically by the IDEAS software (Luminex ver. 6.2) to create a compensation matrix.

**Figure 1:**
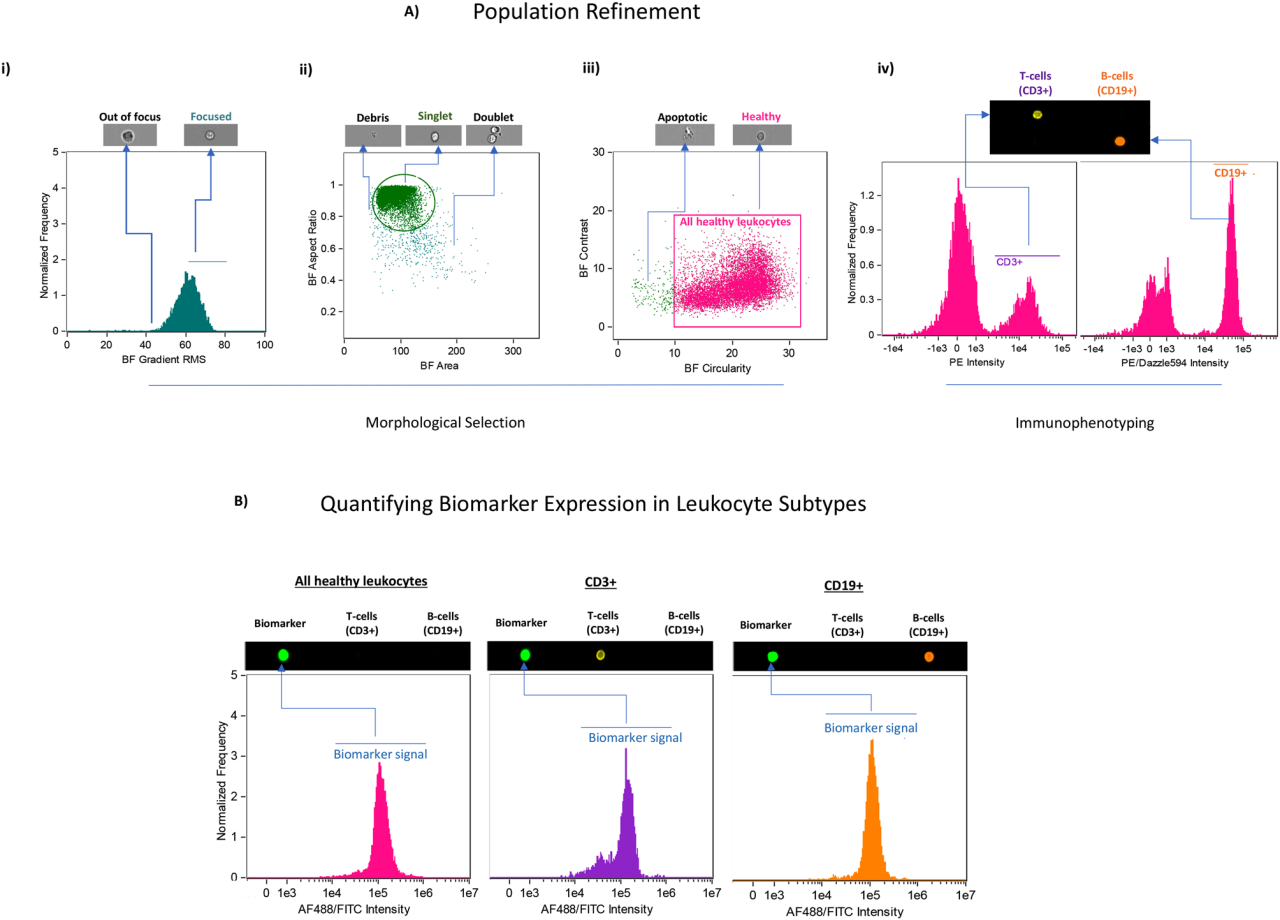
Representative example of our approach for analysis of imaging flow cytometry images and spectral data using IDEAS software. (**A**) Population Refinement. The template first gates for focused, single, healthy cells in T and B populations as follows: (Ai) to select only focused cells for analysis, images of cells were visually inspected, and a relevant region was set on the brightfield (BF) Gradient root mean square (RMS) feature. (Aii) Single cells (singlets) were selected by creating a gate in a bivariate plot of BF Aspect Ratio versus BF Area. (Aiii) Healthy cells were selected by creating a gate in bivariate plot of BF Circularity versus BF Contrast, thus excluding apoptotic cells. (Aiv) B and T cell populations were selected using regions for CD19 + and CD3 + signals, respectively. (**B**) Quantifying Biomarker Expression in Leukocyte Subtypes. The mean fluorescence intensity (MFI) of each biomarker signal within all healthy leukocytes, CD3 + , and CD19 + cell populations was then computed by the IDEAS software. This analysis template was applied to all data files and automatically batch processed within IDEAS.

Analysis imaging flow cytometry images and spectral data were performed on IDEAS software (version 6.2), similar to previous work done in our laboratory^22^. As seen in Fig. 1, we developed a uniform analysis template to quantify the Mean Fluorescence Intensity (MFI) of each biomarker in non-apoptotic mouse leukocytes, CD19 + (B cell) and CD3 + (T cell) populations. Figure 1A illustrates our cell gating methods, as follows: To select only focused cells for analysis, images of cells were visually inspected, and a region with X coordinate beginning at 57.87 was set on the brightfield (BF) Gradient root mean square (RMS) feature (Fig. 1 Ai). Single cells were selected by creating a gate in a bivariate plot of BF Aspect Ratio versus BF Area (Fig. 1 Aii). Healthy cells were selected by creating a gate in bivariate plot of BF Circularity versus BF Contrast, thus excluding apoptotic cells (Fig. 1 Aiii). Regions CD19 + and CD3 + were created to select for B and T cells, respectively (Fig. 1 Aiv). The Mean of Fluorescence Intensity (MFI) value of each biomarker within all healthy leukocytes, CD3 + , and CD19 + cell populations was then computed by the IDEAS software (Fig. 1B). This analysis template was applied to all data files and automatically batch processed within IDEAS.

#### Combining conventional flow cytometry and IFC data

As described above, we quantified the leukocyte subtypes, using two samples from the same mouse: From the first sample, we obtained raw concentration values from interrogating surface labeled fresh whole blood via conventional flow cytometry (“ln_Bcells / ln_T_cells”, as described in the methods). Later, a second sample was prepared involving fixing, permeabilizing, and multiple washes, from which we obtained percentages of surface labeled subtypes present in the total number cells analyzed on the IFC (“Percent_T cells / Percent_B cells”, as described in the methods). Due to the inherent differences in the preparation methods for these pre- and post- processed samples and the capabilities of the instrument they were interrogated with, each sample generated data with different metrics: The pre-processed raw (non-fixed) counts measured by conventional flow cytometry are likely to provide values that more closely reflect absolute cell numbers in the sample. These numbers represent exponential cell killing by radiation and are therefore log-transformed. In contrast, the IFC-prepared samples undergo several processing steps towards the measurement of intracellular and surface biomarker labeling of the B and T cell subtypes, all of which are based on brightfield morphology and refined by several image gating steps (as seen in Fig. 1). Therefore, it is of interest to look at both methods of quantifying blood counts in determining correlation with radiation dose and exposure type (Interaction).

### Data set for machine learning analyses

Biomarker signals and conventional flow cytometry blood counts were natural log (ln) transformed to bring their distributions closer to the normal distribution. The main variables in the resulting data set were: The radiation dose (Dose, in Gy). The exposure type (Exposure), with 1.0 for TBI and 0.5 for PBI. The product of dose and exposure type (Interaction), with 1.0 × Dose for TBI and 0.5 × Dose for PBI. Sex, with 0 = females and 1 = males. Radiation type (Radiation_type), with 0 for electrons and 1 for x-rays. Ln-transformed B and T cell counts (ln_B_cells and ln_T_cells, respectively) from CytoFLEX measurements are given in events/µl. Percentages of cells displaying CD3 or CD19 surface markers (Percent_T cells and Percent_B cells, respectively) from IFC measurements are given as a percentage of all healthy, single cells which were analyzed. Ln-transformed signals (from the Intensity_MC_Ch02, Mean, healthy & single & focused channel) for the radiation-responsive biomarkers. This data set is provided in Supplementary_table_S1 online. Dose and Interaction were treated as the target variables to be predicted by the ML models, using the other variables (except Exposure) as predictors.

### Machine learning analysis procedure

We imported the data into the *R* 4.2.0^41^ programming language. We used the geometric mean of unstained blood samples from each batch to normalize biomarker fluorescence intensities and reduce potential differences in signal intensities between experimental batches (*i.e.,* groups of mice irradiated on the same day). Analyses and visualizations of the data were performed in *R* and in Microsoft Excel software.

We split the data set randomly into halves for training and testing. We used the Boruta feature selection algorithm (implemented by the *Boruta R* package)^42^ to identify and discard any weak predictor variables, which would not be useful for reconstructing the Dose or Interaction variables. Boruta iteratively compares the importance score of each predictor with the importance score of its randomly shuffled “shadow”, in the context of a random forest model^42^. It duplicates the data set and randomly shuffles the values in each column. These shuffled values are called shadow features, and they are re-created in each iteration. Those predictors that had significantly (*p*-value < 0.05 with Bonferroni correction) worse importance than shadow features during Boruta implementation on a randomly selected training half of the data were discarded from further analysis.

We trained the random forest (RF) ML algorithm^43^ on the training portion of the data set, using all predictor variables retained by Boruta, to predict Dose or Interaction. Each of these RF models was refined by grid search hyperparameter tuning, using the *caret* and *ranger R* packages, separately for each of the two target variables. In addition, we trained a separate RF model in classification mode to distinguish between exposed and unexposed samples (*i.e.* those with radiation Dose > 0 versus 0).

The strengths of the RF algorithm include its ability to model non-linear relationships and interactions between variables, and its low sensitivity to correlations between predictor variables and to outlier observations^43^. RF generates many uncorrelated decision trees by bootstrap aggregation, or “bagging” (randomly selecting samples from training data with replacement) and feature randomness (selecting a random subset of predictor variables for each tree). Predictions from all trees are then averaged for regression problems such as the one here.

To counteract the problem of overfitting, we trained each RF model using repeated *k*-fold cross validation (threefold, repeated 100 times) on the training data, and evaluated its performance on the testing data. Three performance metrics were used for evaluation on each of the target variables (Dose or Interaction): mean absolute error (MAE), root mean square root error (RMSE) and coefficient of determination (R^2^).

## Results

### Biomarker and blood cell count dose responses for PBI and TBI

**Figure 2:**
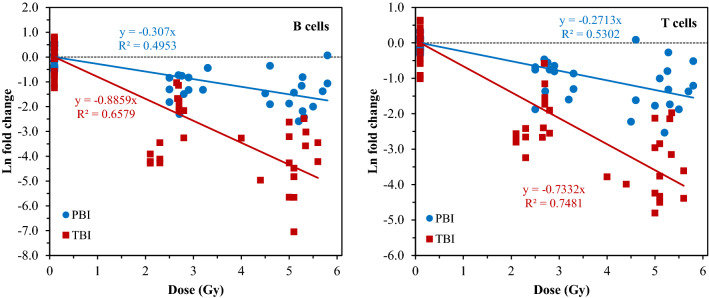
Radiation dose responses (ln-transformed fold changes relative to unexposed mice) for B and T cells. In this and the following figures, scatter along the x-axis represents the spread of individual mouse dosimetry estimates, which varied somewhat around the nominally prescribed doses. The data are “raw” counts from flow cytometry. The black dotted line represents zero ln fold change and indicates the baseline.

The dose responses for B and T cell counts obtained by conventional flow cytometry after TBI or PBI exposures are shown in Fig. 2. Overall, these results show that despite some variability between different mice, it is clear that the dose response slopes were markedly different for TBI (red) and PBI (blue) exposures. The linear regression analysis which generated the fitted lines in Fig. 2 showed that the PBI slopes were roughly twofold lower than TBI slopes, reflecting that PBI was half-body in this case and that the differences in slopes between TBI and PBI were statistically significant: *p*-value = 6.13 × 10^−7^ for B cells and 2.65 × 10^−8^ for T cells. In each case, the null hypothesis was that the regression slopes are equivalent for PBI versus TBI. Coefficient of determination (R^2^) values are also shown in Fig. 2. These values (especially for TBI, 0.66 and 0.75 for B and T cells, respectively) suggested that most of the data variability was explained by the linear regression.

**Figure 3:**
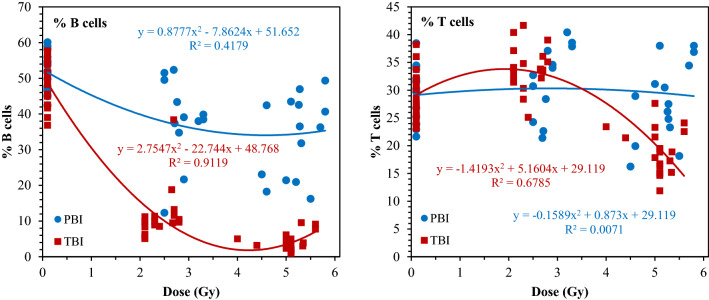
Radiation dose responses for the percentage of cells that display B or T cell surface markers, among all single, focused, and non-apoptotic cells that were acquired. The data shown are "post-processed" counts from IFC.

The TBI and PBI dose responses of the percentages of T and B cells that met the sequential gating criteria by IFC shown in Fig. 3. As described in the Methods section, these percentages represent a different metric, than the raw B and T cell counts shown in Fig. 2. The difference in the shapes of the curves in Figs. 2 and 3 may be due to the different methods in sample preparation: the raw counts (non-fixed) measured by conventional flow cytometry are more representative of a total population of healthy and dying cells, whereas the IFC-processed fixed/permeabilized samples gated for healthy T and B leukocyte subtypes were used to estimate in more detail how the percentages of different cell populations with different surface markers changed as a function of radiation dose and type. Importantly, for both types of metrics the dose responses looked considerably different for PBI versus TBI exposures. Consequently, both the raw B and T cell counts and the percentages of B and T leukocyte subtypes from IFC were incorporated as predictor variables into the dose reconstruction ML modeling.

**Figure 4:**
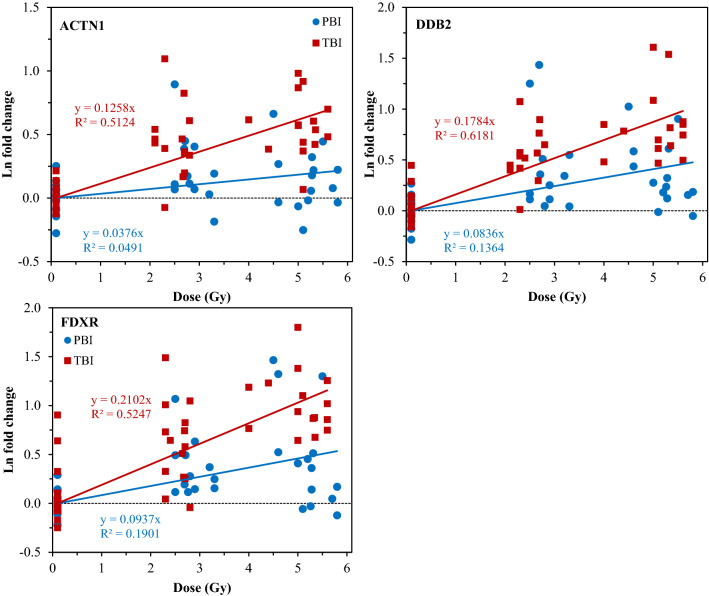
Radiation dose responses (ln-transformed fold changes relative to unexposed mice) for three protein biomarkers (ACTN1, DDB2, FDXR) after TBI or PBI. The black dotted line represents zero ln fold change and indicates the baseline.

The measured radiation dose responses for the protein biomarkers DDB2, FDXR and ACTN1 are shown in Fig. 4. Here the ln-transformed fold changes are increasing with dose instead of decreasing, but also there is a clear and statistically significant difference in dose response slopes between TBI and PBI: *p*-value = 1.20 × 10^−3^ for DDB2, 5.09 × 10^−4^ for FDXR, and 3.02 × 10^−4^ for ACTN1. The dose response slopes for PBI are approximately twofold lower than the corresponding TBI values, which supports the expectation that approximately half of the body was irradiated in the PBI scenario. The DDB2 and FDXR biomarkers showed the most reproducible dose response patterns among experimental batches, and therefore we focused on measuring these two biomarkers in the subsequent ML analyses.

### Selection of strong predictors of radiation dose and exposure type

**Figure 5:**
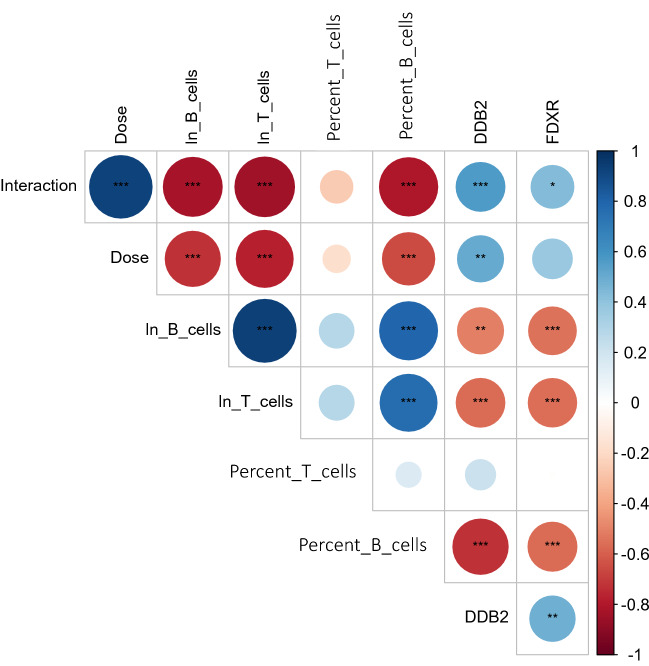
Matrix of Spearman’s correlation coefficients between all variables in the training data. Interaction = Dose for TBI, and Interaction = Dose/2 for PBI. Blue circles represent positive correlations, and red circles represent negative ones. Larger circles represent stronger correlations. Asterisk symbols represent Bonferroni-corrected *p*-values: * < 0.05, ** < 0.01, *** < 0.001.

As described in Materials and Methods, the data set was split randomly into training and testing halves. The training part was used for feature selection (*i.e.,* identifying the most important predictors of Dose and Interaction), tuning and fitting of the RF model or each target variable. The testing part was used to evaluate model performance. A visualization of the matrix of Spearman’s correlation coefficients between all variables (*e.g.,* blood cell counts, biomarkers) in the training data is displayed in Fig. 5. It shows that many of the predictor variables were strongly correlated with the outcome variables, Dose and/or Interaction. The B and T cell counts were very strongly correlated with the outcome variables, and the selected protein biomarkers (especially DDB2) showed significant correlations as well.

To determine which predictor variables are most important and need to be retained for ML analysis, we implemented the Boruta feature selection algorithm^42^. Each predictor is retained only if it outperforms its “shadow” with a specified level of statistical significance (here set to 0.05 with Bonferroni correction). In this case, the Sex and Radiation_type variables did not pass the Boruta screening, suggesting that they are not very important for reconstruction of Dose or Interaction (where Interaction = Dose for TBI, and Interaction = Dose/2 for PBI). Specifically, Sex and Radiation_type outperformed noise in only 0—16.7% of Boruta iterations, whereas the other predictor variables (ln_B_cells, ln_T_cells, Percent_T cells, Percent_B cells, DDB2 and FDXR) did so in 87.5–100% of iterations. Therefore, the data from mice irradiated with TBI x-rays were not distinguished by Boruta screening from those data that came from electron-exposed TBI mice, which is biologically plausible since low-LET photons and electrons tend to have similar biological effectiveness per unit dose^44^.

This finding of similarity between electron and x-ray effects in this study is supported by calculation of dose reconstruction performance metrics for testing TBI samples, separately for electron and x-ray irradiations. For electrons, R^2^ = 0.949, RMSE = 0.539 Gy, and MAE = 0.413 Gy. For x-rays, the numbers were quite similar: R^2^ = 0.962, RMSE = 0.501 Gy, and MAE = 0.403 Gy.

### Machine learning results for dose and exposure type reconstructions

In this analysis, Interaction = Dose for TBI exposures, and Interaction = Dose/2 for PBI exposures. Two separate random forest models were fitted to the data. The set of predictor variables was the same for each model (ln_B_cells, ln_T_cells, Percent_T cells, Percent_B cells, DDB2, DXR), but the target variable to be predicted was different: Dose in one model, and Interaction in the other. The rationale for this approach was that in a hypothetical realistic situation where samples with unknown exposures are analyzed, both models will be used and predictions for both Dose and Interaction will be generated for each sample. This dual prediction is intended to be informative about the type and magnitude of exposure for the sample.

**Figure 6:**
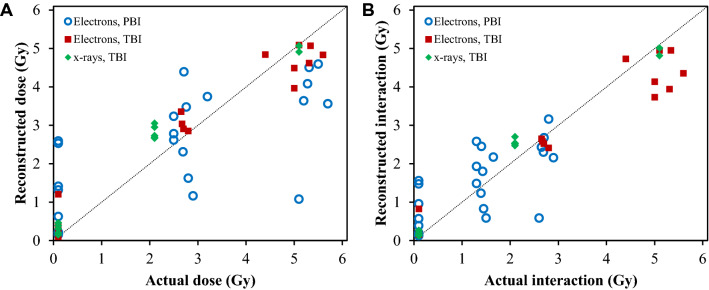
(**A**) Actual versus reconstructed Dose on testing data, based on the RF algorithm. Overall R^2^ = 0.738, root mean squared error (RMSE) = 1.060 Gy, mean absolute error (MAE) = 0.749 Gy. Among the 0 Gy samples, RMSE = 0.978 Gy and MAE = 0.601 Gy. Among the 2.0–2.5 Gy samples, RMSE = 0.824 Gy and MAE = 0.684 Gy. Among the 5.0 Gy samples, RMSE = 1.385 Gy and MAE = 0.971 Gy. (**B**) Actual versus reconstructed Interaction (= Dose/2 for PBI, = Dose for TBI). Overall R^2^ = 0.868, RMSE = 0.663 Gy, MAE = 0.472 Gy. Among the 0 Gy samples, RMSE = 0.560 Gy and MAE = 0.332 Gy. Among the 2.0–2.5 Gy samples, RMSE = 0.677 Gy and MAE = 0.516 Gy. Among the 5.0 Gy samples, RMSE = 0.826 Gy and MAE = 0.665 Gy.

The tuned RF models based on the 6 retained predictors used all 6 (mtry = 6), with a minimum number of samples in a node of 1 (min.node.size = 1), for each outcome variable (Dose or Interaction). For predicting Dose, the predictor rankings (from most to least important) were: ln_T_cells, Percent_B cells, ln_B_cells, Percent_T cells, DDB2, FDXR. For predicting Interaction, the predictor rankings (from most to least important) were: Percent_B cells, ln_T_cells, ln_B_cells, Percent_T cells, DDB2, FDXR. The results of RF performance, which compare actual with reconstructed values of Dose and Interaction, are shown in Fig. 6. For Dose (Fig. 6A), R^2^ = 0.738, RMSE = 1.060 Gy, MAE = 0.749 Gy. For Interaction (Fig. 6B), R^2^ = 0.868, RMSE = 0.663 Gy, MAE = 0.472 Gy. Notably, these reconstructions were reasonably accurate (Fig. 6) despite inter-individual variability between mice. All RF predictions on testing data are provided in Supplementary_table_2 online.

**Figure 7:**
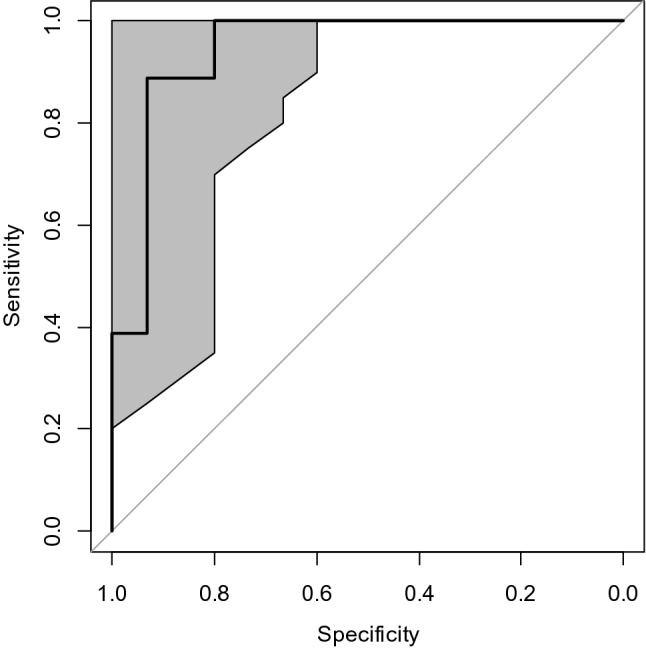
Discrimination between PBI and TBI exposures by the RF algorithm among irradiated samples in the testing data set. We used predicted Dose—predicted Interaction to predict TBI versus PBI status for each irradiated sample. The ROC curve AUC for this analysis = 0.944 (95% CI: 0.844–1.0). The grey shaded areas represent 95% confidence regions estimated by 10,000 bootstrap replicates. The diagonal black lines represent random classification performance.

Among irradiated mouse samples in the testing data set, it was possible to discriminate between PBI and TBI by predicting the Dose – Interaction difference (Fig. 7). Here, only irradiated animal data was used, and un-irradiated controls were excluded. Predicted values of Dose and Interaction, which were calculated by RF models as described above, were used to calculate the predicted Dose-Interaction difference for each irradiated sample. This difference was used to classify samples into the TBI or PBI classes, and classification results were compared with true known values of TBI or PBI for each sample to generate the ROC curve shown in Fig. 7. These results suggest that ML-based methods can be useful for detecting PBI exposures based on protein biomarker and blood cell count data as inputs. In addition, despite the variability in responses between individual mice, since the RF algorithm integrates information from several predictors (B and T cell counts and percentages, FDXR and DDB2 biomarkers), it was able to accurately classify samples as exposed or unexposed: classification accuracy on the testing data set was 92.2% and ROC curve AUC = 0.982 (95% CI: 0.953, 1.0). The comparisons of data with RF predictions are provided in Supplementary_table_S3 online.

## Discussion

The objective of this study was to investigate the usefulness of radiation responsive protein biomarkers, in combination with blood cell counts, as potential rapid and high-throughput biodosimeters for PBI as well as TBI exposure situations. Enhancing the number of available tools for PBI biodosimetry is important because currently available techniques have limitations in terms of time-to-result, throughput and/or accuracy. We hypothesized that combining radiation-responsive protein biomarkers and blood cell counts in an ML model context can be used to generate quantitative reconstructions of the radiation dose for PBI as well as for TBI exposures. The results suggest that the top two intracellular protein biomarker expression (DDB2, FDXR), and immunophenotyping through either traditional flow (cell counts) or IFC (cell percentages after gating) correlated strongly with radiation exposure (Fig. 5), and showed consistent and reproducible dose-dependent radiation responses (Figs. 2, 3, 4). The slopes of these responses for biomarkers and blood cell counts were significantly different for TBI versus PBI irradiated mice. These findings support the expectation that PBI exposures “spare” a large fraction of blood cells from radiation damage. Based on these differences, an ML analysis using the RF algorithm was able to generate accurate reconstructions of PBI exposures, as well as TBI. The RF algorithm was also able to distinguish between unirradiated and irradiated samples. Consequently, it may be possible in the future to use separate RF models in a two-stage process to first classify each unknown sample as either irradiated or unirradiated, and then to distinguish TBI from PBI on those samples classified as irradiated.

It is important to emphasize that quantification of radiation dose, as well as classification of the exposure type as TBI versus PBI, are important first steps, but ultimately they need to be followed by much more detailed assessment of the possible acute and long-term health effects of the exposure. In other words, predicting the likely symptoms of irradiation and taking the correct actions to prevent and/or mitigate them is the ultimate goal, where dose and exposure type reconstruction are the initial steps.

We believe that the results of this study are the first use of intracellular protein and cell surface biomarkers for biodosimetry in an ML context and support the potential usefulness of the proposed approach for biodosimetry in practical mass-exposure situations, such as improvised nuclear device explosion scenarios, for time points soon after the event (*e.g.,* 24 h), as well as for longer time points. However, limitations of the current study include selection of one age group only (young adult), a single (half-body) PBI shielding setup, and only two non-zero dose levels. Other limitations include the use of a single ML method (RF) and, ultimately, the challenges of translatability from the mouse system to humans. Also, the classification of exposures into TBI versus PBI categories, as performed here, is a simple “extreme” representation of a more complex picture of inhomogeneous radiation exposures, which was used here mainly as a proof of principle to develop/refine biodosimetry methods.

We are planning to address the first three of these limitations by acquiring young (4 week old) mice, using a hind leg shielding set up (which shields only a small percentage of the bone marrow), and investigating the performances of other state of the art ML methods, such as extreme gradient boosting (XGBoost)^45^, to improve the dose and exposure reconstructions. The age issue is particularly important since radiosensitivity, for example carcinogenesis, can be higher in pediatric populations than in adults^46,47^. We plan to assess whether or not the same exposure reconstruction approaches and choice of predictors are applicable to young mice as well as to adults, or whether different approaches and biomarkers are needed for different age groups.

In summary, we are developing a biomarker-based FAST-DOSE biodosimetry assay that can be used to rapidly quantify intracellular and surface protein markers to accurately estimate absorbed dose after exposure to TBI and PBI. The current study shows that this approach can distinguish between PBI and TBI exposures and quantify them, but this was only a first step, which used a limited number of dose levels and a single – half-body – PBI exposure condition. The development of an in-the-field FAST-DOSE biodosimeter for estimation of absorbed radiation dose in potentially exposed individuals shortly after radiation exposure would allow for rapid triage and treatment decisions prior to sending blood samples for more accurate cytogenetic testing^32,33^. In future work, we plan to further validate this system using more doses and PBI exposure scenarios, and to optimize its performance for time points up to a week after radiation exposure and to transition the top biomarker candidates to an in-the-field deployable device.

## Supplementary Information

Supplementary Information 1.

Supplementary Information 2.

Supplementary Information 3.

## Data Availability

All datasets analyzed during the current study are available in Supplementary_table_S1 online and also available from corresponding author on reasonable request.
