# Supplementary material for: Machine learning approach for quantitative biodosimetry of partial-body or total-body radiation exposures by combining radiation-responsive biomarkers
Source: Sci Rep. 2023 Jan 18;13:949. doi: 10.1038/s41598-023-28130-0 (PMC9849198; doi:10.1038/s41598-023-28130-0)
Supplement: Supplementary file 1 — Supplementary Information 1. [file 41598_2023_28130_MOESM1_ESM.pdf]

**Supplementary table S1. This is the complete data set analyzed in the current study. The r variable is described in the main text.**

| Dose | Exposure | Interaction | Sex | Radiation_type | ln_B_cells | ln_T_cells | Percent_CD3 | Percent_CD19 |
|---|---|---|---|---|---|---|---|---|
| 0 | 0.5 | 0 | 1 | 0 | 4.7362 | 5.0626 | 26.8251 | 54.305 |
| 0 | 0.5 | 0 | 1 | 0 | 4.60517 | 4.79579 | 23.93 | 58.0664 |
| 0 | 0.5 | 0 | 1 | 0 | 3.97029 | 4.5326 | 22.9592 | 58.9456 |
| 0 | 0.5 | 0 | 1 | 0 | 3.89182 | 4.44265 | 25.2327 | 60.1085 |
| 2.4 | 0.5 | 1.2 | 1 | 0 | 2.99573 | 3.95124 | 30.7352 | 49.5483 |
| 2.4 | 0.5 | 1.2 | 1 | 0 | 2.48491 | 2.83321 | 32.7152 | 12.3255 |
| 2.4 | 0.5 | 1.2 | 1 | 0 | 3.46574 | 4.02535 | 24.2539 | 51.5211 |
| 4.4 | 0.5 | 2.2 | 1 | 0 | 2.83321 | 2.48491 | 16.2387 | 23.0554 |
| 4.5 | 0.5 | 2.25 | 1 | 0 | 2.3979 | 3.09104 | 19.9377 | 18.2435 |
| 4.5 | 0.5 | 2.25 | 1 | 0 | 3.95124 | 4.79579 | 28.9336 | 42.4254 |
| 5.4 | 0.5 | 2.7 | 1 | 0 | 2.30259 | 2.83321 | 18.1602 | 16.2475 |
| 0 | 0.5 | 0 | 1 | 0 | 4.91998 | 5.32788 | 23.8945 | 52.0347 |
| 0 | 0.5 | 0 | 1 | 0 | 4.63473 | 4.91998 | 23.8473 | 55.8983 |
| 0 | 0.5 | 0 | 1 | 0 | 5.65599 | 4.84419 | 21.6368 | 54.8422 |
| 2.59 | 0.5 | 1.295 | 1 | 0 | 4.34381 | 4.56435 | 21.3989 | 52.3546 |
| 2.61 | 0.5 | 1.305 | 1 | 0 | 2.77259 | 3.66356 | 22.6364 | 37.3877 |
| 2.66 | 0.5 | 1.33 | 1 | 0 | 4.31749 | 4.47734 | 28.3955 | 43.3391 |
| 5.16 | 0.5 | 2.58 | 1 | 0 | 3.91202 | 4.23411 | 26.1486 | 42.5391 |
| 5.18 | 0.5 | 2.59 | 1 | 0 | 4.26268 | 4.76217 | 27.5097 | 46.9681 |
| 5.18 | 0.5 | 2.59 | 1 | 0 | 2.89037 | 3.71357 | 24.8306 | 36.5201 |
| 5.21 | 0.5 | 2.605 | 1 | 0 | 2.63906 | 3.29584 | 23.3053 | 31.8013 |
| 0 | 0.5 | 0 | 0 | 0 | 4.74493 | 4.91998 | 38.4622 | 42.3917 |
| 0 | 0.5 | 0 | 0 | 0 | 4.2485 | 4.85981 | 33.7871 | 44.9606 |
| 0 | 0.5 | 0 | 0 | 0 | 4.17439 | 4.8828 | 34.3232 | 45.1664 |
| 3.2 | 0.5 | 1.6 | 0 | 0 | 3.71357 | 3.8712 | 37.8911 | 39.8451 |
| 3.2 | 0.5 | 1.6 | 0 | 0 | 3.71357 | 3.43399 | 38.576 | 38.496 |
| 3.1 | 0.5 | 1.55 | 0 | 0 | 2.83321 | 3.13549 | 40.4307 | 38.0125 |
| 5.7 | 0.5 | 2.85 | 0 | 0 | 4.21951 | 3.52636 | 36.8756 | 49.3644 |
| 5.7 | 0.5 | 2.85 | 0 | 0 | 3.09104 | 4.21951 | 38.003 | 40.6693 |
| 5.6 | 0.5 | 2.8 | 0 | 0 | 2.77259 | 3.3673 | 34.441 | 36.2793 |
| 0 | 0.5 | 0 | 0 | 0 | 3.98898 | 4.60517 | 32.1097 | 46.5293 |
| 0 | 0.5 | 0 | 0 | 0 | 3.61092 | 4.40672 | 31.4727 | 51.624 |
| 0 | 0.5 | 0 | 0 | 0 | 6.68336 | 5.88332 | 29.667 | 53.6714 |
| 0 | 0.5 | 0 | 0 | 0 | 6.29157 | 5.95064 | 34.4587 | 49.845 |
| 0 | 0.5 | 0 | 0 | 0 | 6.05912 | 5.63121 | 31.6229 | 47.6859 |
| 2.8 | 0.5 | 1.4 | 0 | 0 | 5.37528 | 5.0626 | 34.0299 | 21.6684 |
| 2.8 | 0.5 | 1.4 | 0 | 0 | 4.8752 | 5.22036 | 34.5916 | 39.1088 |
| 2.7 | 0.5 | 1.35 | 0 | 0 | 4.70953 | 5.11199 | 37.0881 | 34.7657 |
| 5.1 | 0.5 | 2.55 | 0 | 0 | 3.61092 | 3.3322 | 30.4896 | 20.9568 |
| 4.9 | 0.5 | 2.45 | 0 | 0 | 4.33073 | 4.09434 | 31.1176 | 21.4574 |

| | | | | | | | | |
|---|---|---|---|---|---|---|---|---|
| 5 | 0.5 | 2.5 | 0 | 0 | 4.77068 | 4.85981 | 38.0037 | 43.4574 |
| 0 | 0.5 | 0 | 0 | 0 | 5.77765 | 5.99645 | 30.273 | 46.8715 |
| 0 | 1 | 0 | 1 | 0 | 7.22111 | 6.1717 | 23.1004 | 56.9158 |
| 0 | 1 | 0 | 1 | 0 | 7.11883 | 6.44731 | 23.6985 | 57.5559 |
| 0 | 1 | 0 | 1 | 0 | 5.91889 | 5.93754 | 26.1686 | 57.352 |
| 2.2 | 1 | 2.2 | 1 | 0 | 2.63906 | 3.52636 | 34.8097 | 11.3583 |
| 2.2 | 1 | 2.2 | 1 | 0 | 2.48491 | 2.94444 | 30.3347 | 9.80293 |
| 2.7 | 1 | 2.7 | 1 | 0 | 3.49651 | 3.63759 | 39.0398 | 9.70417 |
| 4.3 | 1 | 4.3 | 1 | 0 | 1.79176 | 2.19722 | 21.4322 | 3.19293 |
| 4.9 | 1 | 4.9 | 1 | 0 | 2.48491 | 1.94591 | 27.6257 | 3.28973 |
| 4.9 | 1 | 4.9 | 1 | 0 | 1.09861 | 1.38629 | 17.8482 | 2.36163 |
| 0 | 1 | 0 | 1 | 0 | 5.273 | 5.54518 | 23.4646 | 55.3314 |
| 0 | 1 | 0 | 1 | 0 | 5.23644 | 5.36129 | 25.3103 | 51.0497 |
| 0 | 1 | 0 | 1 | 0 | 4.39445 | 4.78749 | 24.6417 | 51.5022 |
| 2.55 | 1 | 2.55 | 1 | 0 | 3.93183 | 2.56495 | 33.7927 | 18.7732 |
| 2.57 | 1 | 2.57 | 1 | 0 | 3.29584 | 2.83321 | 32.1805 | 9.509 |
| 2.59 | 1 | 2.59 | 1 | 0 | 3.82864 | 4.65396 | 33.5809 | 38.361 |
| 2.6 | 1 | 2.6 | 1 | 0 | 2.94444 | 4.07754 | 36.0892 | 13.253 |
| 5.21 | 1 | 5.21 | 1 | 0 | 2.48491 | 3.09104 | 17.2819 | 9.56523 |
| 5.24 | 1 | 5.24 | 1 | 0 | 1.38629 | 2.07944 | 15.2082 | 3.01862 |
| 5.25 | 1 | 5.25 | 1 | 0 | 1.94591 | 3.2581 | 18.8891 | 3.90858 |
| 0 | 1 | 0 | 0 | 0 | 6.87213 | 6.82002 | 33.7212 | 41.6736 |
| 0 | 1 | 0 | 0 | 0 | 6.17794 | 6.45205 | 36.0513 | 45.5739 |
| 0 | 1 | 0 | 0 | 0 | 5.42935 | 5.2575 | 38.2252 | 42.2762 |
| 2.2 | 1 | 2.2 | 0 | 0 | 2.70805 | 3.7612 | 41.6575 | 8.911 |
| 3.9 | 1 | 3.9 | 0 | 0 | 2.89037 | 2.3979 | 23.4199 | 5.03493 |
| 5.5 | 1 | 5.5 | 0 | 0 | 2.70805 | 2.56495 | 24.1136 | 7.7476 |
| 5.5 | 1 | 5.5 | 0 | 0 | 1.94591 | 1.79176 | 22.548 | 9.11637 |
| 0 | 1 | 0 | 0 | 0 | 6.27099 | 6.06379 | 31.5236 | 51.7571 |
| 0 | 1 | 0 | 0 | 0 | 6.73221 | 6.41346 | 27.6654 | 51.0841 |
| 0 | 1 | 0 | 0 | 0 | 5.85507 | 5.9108 | 31.9744 | 48.2631 |
| 2.7 | 1 | 2.7 | 0 | 0 | 3.7612 | 3.89182 | 35.0942 | 10.4619 |
| 2.6 | 1 | 2.6 | 0 | 0 | 3.7612 | 4.06044 | 33.6357 | 12.9906 |
| 2.6 | 1 | 2.6 | 0 | 0 | 4.15888 | 4.2485 | 33.4476 | 11.6141 |
| 4.9 | 1 | 4.9 | 0 | 0 | 2.70805 | 2.83321 | 21.5614 | 6.13763 |
| 4.9 | 1 | 4.9 | 0 | 0 | 3.29584 | 3.66356 | 23.2988 | 4.3573 |
| 5 | 1 | 5 | 0 | 0 | 1.09861 | 2.94444 | 16.3205 | 2.2575 |
| 0 | 1 | 0 | 0 | 0 | 4.82028 | 4.77912 | 32.0359 | 45.2066 |
| 0 | 1 | 0 | 0 | 1 | 7.59438 | 7.03527 | 26.975 | 36.8479 |
| 0 | 1 | 0 | 0 | 1 | 7.79647 | 7.32185 | 26.9609 | 48.9638 |
| 0 | 1 | 0 | 0 | 1 | 6.98749 | 6.91274 | 28.5458 | 39.0223 |
| 0 | 1 | 0 | 0 | 1 | 5.80513 | 6.31355 | 30.1655 | 42.562 |
| 2 | 1 | 2 | 0 | 1 | 2.83321 | 4.09434 | 37.1239 | 5.1006 |
| 2 | 1 | 2 | 0 | 1 | 3.13549 | 4.33073 | 31.902 | 5.8829 |
| 2 | 1 | 2 | 0 | 1 | 2.77259 | 4.26268 | 34.0915 | 5.24623 |
| 5 | 1 | 5 | 0 | 1 | 2.56495 | 3.13549 | 18.872 | 3.6686 |
| 5 | 1 | 5 | 0 | 1 | 0 | 2.3979 | 14.6689 | 1.55303 |

| | | | | | | | | |
|---|---|---|---|---|---|---|---|---|
| 5 | 1 | 5 | 0 | 1 | 1.38629 | 2.56495 | 16.7313 | 1.7308 |
| 0 | 1 | 0 | 1 | 1 | 7.87017 | 6.83518 | 25.4424 | 57.6933 |
| 0 | 1 | 0 | 1 | 1 | 7.42952 | 6.94698 | 31.0586 | 50.3766 |
| 0 | 1 | 0 | 1 | 1 | 6.62407 | 6.49979 | 27.4077 | 53.9195 |
| 0 | 1 | 0 | 1 | 1 | 5.04343 | 5.77144 | 32.2345 | 51.2795 |
| 0 | 1 | 0 | 1 | 1 | 4.70048 | 5.6058 | 31.4565 | 44.9348 |
| 2 | 1 | 2 | 1 | 1 | 2.48491 | 3.52636 | 31.4351 | 6.27597 |
| 2 | 1 | 2 | 1 | 1 | 2.99573 | 3.97029 | 40.3991 | 8.38297 |
| 2 | 1 | 2 | 1 | 1 | 2.70805 | 3.52636 | 33.081 | 9.8098 |
| 2 | 1 | 2 | 1 | 1 | 2.89037 | 2.99573 | 33.88 | 9.81403 |
| 2 | 1 | 2 | 1 | 1 | 3.09104 | 2.99573 | 34.0204 | 11.226 |
| 5 | 1 | 5 | 1 | 1 | 0 | 2.19722 | 19.5045 | 1.84457 |
| 5 | 1 | 5 | 1 | 1 | 2.07944 | 2.48491 | 19.488 | 5.0015 |
| 5 | 1 | 5 | 1 | 1 | 0 | 2.3979 | 15.1037 | 1.21617 |
| 5 | 1 | 5 | 1 | 1 | 0 | 1.09861 | 11.873 | 0.96993 |
| 5 | 1 | 5 | 1 | 1 | 2.48491 | 2.19722 | 14.9974 | 2.06567 |

**neaning of each**

| DDB2 | FDXR |
|---|---|
| -0.533 | 1.99205 |
| -0.6666 | 1.76196 |
| -0.6568 | 1.8114 |
| -0.6794 | 1.82798 |
| -0.4668 | 2.34161 |
| 0.61674 | 2.91675 |
| -0.5213 | 1.96503 |
| 0.39096 | 3.3135 |
| -0.0487 | 3.17096 |
| -0.1978 | 2.3718 |
| 0.27 | 3.14856 |
| -0.5025 | 0.7606 |
| -0.566 | 0.91008 |
| -0.324 | 1.27162 |
| 0.97061 | 1.17439 |
| -0.1068 | 1.47456 |
| 0.04449 | 1.09672 |
| -0.2276 | 0.95188 |
| -0.1412 | 1.34262 |
| -0.3417 | 1.12281 |
| 0.14747 | 1.49374 |
| 0.95211 | 1.99239 |
| 0.69614 | 1.86895 |
| 0.62812 | 1.78913 |
| 0.72818 | 2.11543 |
| 1.23471 | 2.02239 |
| 1.02866 | 2.23805 |
| 0.63469 | 1.74543 |
| 0.87159 | 2.03737 |
| 0.84157 | 1.91456 |
| 0.74973 | 1.92616 |
| 0.40142 | 1.75964 |
| 0.14937 | 1.58686 |
| 0.48386 | 1.51613 |
| 0.26782 | 1.6401 |
| 0.57662 | 2.22148 |
| 0.43855 | 1.73515 |
| 0.37187 | 1.86737 |
| 0.50602 | 2.04374 |
| 0.60176 | 1.99869 |

| | |
|---|---|
| 0.31334 | 1.53243 |
| 0.40008 | 1.61263 |
| -0.2413 | 2.38272 |
| -0.6663 | 1.97924 |
| -0.6844 | 1.80645 |
| 0.00757 | 3.06461 |
| 0.54278 | 3.5458 |
| 0.1189 | 3.10395 |
| 0.25318 | 3.28679 |
| 0.55534 | 3.43684 |
| 1.07767 | 3.85595 |
| -0.462 | 0.90381 |
| -0.3887 | 0.94492 |
| -0.4518 | 0.93178 |
| 0.13411 | 1.43685 |
| -0.1375 | 1.19442 |
| 0.3299 | 1.67028 |
| 0.46225 | 1.75176 |
| 1.10402 | 1.79511 |
| 0.38269 | 1.80436 |
| 0.20589 | 1.6021 |
| 0.49005 | 1.76904 |
| 0.66041 | 1.80683 |
| 0.79445 | 1.94823 |
| 1.22021 | 2.16829 |
| 1.49699 | 3.02904 |
| 1.52424 | 2.861 |
| 1.39304 | 2.69861 |
| 0.19835 | 1.51981 |
| 0.10471 | 1.51399 |
| 0.12187 | 1.45525 |
| 0.5838 | 2.0773 |
| 0.59213 | 1.75088 |
| 0.63157 | 2.14178 |
| 0.88074 | 2.43593 |
| 0.79479 | 2.59997 |
| 0.65095 | 2.13657 |
| 0.30787 | 1.50193 |
| 0.27171 | 1.78297 |
| 0.13146 | 1.52323 |
| 0.24219 | 1.72955 |
| 0.23151 | 1.73813 |
| 0.73751 | 2.33853 |
| 0.63792 | 2.42418 |
| 0.70084 | 2.45891 |
| 1.06905 | 2.94942 |
| 0.71608 | 2.4422 |

| | |
|---|---|
| 0.66626 | 2.59783 |
| 0.02356 | 1.00787 |
| 0.12368 | 0.88989 |
| 0.03182 | 0.82066 |
| 0.33715 | 0.77864 |
| 0.20394 | 0.97124 |
| 0.62943 | 1.58415 |
| 0.66032 | 1.63608 |
| 0.57956 | 1.58299 |
| 0.37916 | 1.44739 |
| 0.75774 | 1.93061 |
| 0.6606 | 2.15484 |
| 0.66045 | 1.8141 |
| 0.77062 | 1.74879 |
| 0.78724 | 1.8023 |
| 0.75311 | 1.84664 |
