# Supplementary material for: Machine learning approach for quantitative biodosimetry of partial-body or total-body radiation exposures by combining radiation-responsive biomarkers
Source: Sci Rep. 2023 Jan 18;13:949. doi: 10.1038/s41598-023-28130-0 (PMC9849198; doi:10.1038/s41598-023-28130-0)
Supplement: Supplementary file 2 — Supplementary Information 2. [file 41598_2023_28130_MOESM2_ESM.pdf]

**Supplementary table S2. RF model predictions on the testing portion of the data set. The meaning of each v described in the main text.**

| Dose | Interaction | ln_B_cells | ln_T_cells | Percent_CD3 | Percent_CD19 | DDB2 | FDXR | Predicted_Interaction | Predicted_Dose |
|---|---|---|---|---|---|---|---|---|---|
| 0 | 0 | 4.7362 | 5.0626 | 26.8251 | 54.305 | -0.533 | 1.99205 | 0.2874 | 0.527 |
| 2.4 | 1.2 | 2.99573 | 3.95124 | 30.7352 | 49.5483 | -0.4668 | 2.34161 | 1.8311 | 3.1332 |
| 2.4 | 1.2 | 2.48491 | 2.83321 | 32.7152 | 12.3255 | 0.61674 | 2.91675 | 2.478 | 2.6824 |
| 2.4 | 1.2 | 3.46574 | 4.02535 | 24.2539 | 51.5211 | -0.5213 | 1.96503 | 1.3856 | 2.5147 |
| 5.4 | 2.7 | 2.30259 | 2.83321 | 18.1602 | 16.2475 | 0.27 | 3.14856 | 3.0594 | 4.493 |
| 2.59 | 1.295 | 4.34381 | 4.56435 | 21.3989 | 52.3546 | 0.97061 | 1.17439 | 1.133 | 2.209 |
| 2.61 | 1.305 | 2.77259 | 3.66356 | 22.6364 | 37.3877 | -0.1068 | 1.47456 | 2.3492 | 4.2931 |
| 2.66 | 1.33 | 4.31749 | 4.47734 | 28.3955 | 43.3391 | 0.04449 | 1.09672 | 1.7025 | 3.3764 |
| 5.18 | 2.59 | 2.89037 | 3.71357 | 24.8306 | 36.5201 | -0.3417 | 1.12281 | 2.2018 | 3.983 |
| 5.21 | 2.605 | 2.63906 | 3.29584 | 23.3053 | 31.8013 | 0.14747 | 1.49374 | 2.5781 | 4.4049 |
| 0 | 0 | 4.74493 | 4.91998 | 38.4622 | 42.3917 | 0.95211 | 1.99239 | 0.468 | 1.2168 |
| 0 | 0 | 4.17439 | 4.8828 | 34.3232 | 45.1664 | 0.62812 | 1.78913 | 0.8552 | 1.3115 |
| 3.1 | 1.55 | 2.83321 | 3.13549 | 40.4307 | 38.0125 | 1.02866 | 2.23805 | 2.0717 | 3.6488 |
| 5.6 | 2.8 | 2.77259 | 3.3673 | 34.441 | 36.2793 | 0.84157 | 1.91456 | 2.0571 | 3.4606 |
| 0 | 0 | 3.98898 | 4.60517 | 32.1097 | 46.5293 | 0.74973 | 1.92616 | 1.4587 | 2.4919 |
| 0 | 0 | 3.61092 | 4.40672 | 31.4727 | 51.624 | 0.40142 | 1.75964 | 1.3741 | 2.4326 |
| 0 | 0 | 6.68336 | 5.88332 | 29.667 | 53.6714 | 0.14937 | 1.58686 | 0.0285 | 0.0518 |
| 0 | 0 | 6.29157 | 5.95064 | 34.4587 | 49.845 | 0.48386 | 1.51613 | 0.0969 | 0.114 |
| 0 | 0 | 6.05912 | 5.63121 | 31.6229 | 47.6859 | 0.26782 | 1.6401 | 0.0425 | 0.1036 |
| 2.8 | 1.4 | 4.8752 | 5.22036 | 34.5916 | 39.1088 | 0.43855 | 1.73515 | 0.4854 | 1.0639 |
| 2.7 | 1.35 | 4.70953 | 5.11199 | 37.0881 | 34.7657 | 0.37187 | 1.86737 | 0.7269 | 1.524 |
| 5.1 | 2.55 | 3.61092 | 3.3322 | 30.4896 | 20.9568 | 0.50602 | 2.04374 | 2.3295 | 3.5398 |
| 5 | 2.5 | 4.77068 | 4.85981 | 38.0037 | 43.4574 | 0.31334 | 1.53243 | 0.4852 | 0.9773 |
| 0 | 0 | 5.91889 | 5.93754 | 26.1686 | 57.352 | -0.6844 | 1.80645 | 0.1036 | 0.0516 |
| 2.7 | 2.7 | 3.49651 | 3.63759 | 39.0398 | 9.70417 | 0.1189 | 3.10395 | 2.314 | 2.7537 |
| 4.3 | 4.3 | 1.79176 | 2.19722 | 21.4322 | 3.19293 | 0.25318 | 3.28679 | 4.6315 | 4.744 |
| 0 | 0 | 4.39445 | 4.78749 | 24.6417 | 51.5022 | -0.4518 | 0.93178 | 0.7264 | 1.1049 |
| 2.55 | 2.55 | 3.93183 | 2.56495 | 33.7927 | 18.7732 | 0.13411 | 1.43685 | 2.5525 | 3.2571 |
| 2.57 | 2.57 | 3.29584 | 2.83321 | 32.1805 | 9.509 | -0.1375 | 1.19442 | 2.4853 | 2.9371 |
| 5.21 | 5.21 | 2.48491 | 3.09104 | 17.2819 | 9.56523 | 1.10402 | 1.79511 | 3.8435 | 4.5205 |
| 5.24 | 5.24 | 1.38629 | 2.07944 | 15.2082 | 3.01862 | 0.38269 | 1.80436 | 4.8525 | 4.973 |
| 5.5 | 5.5 | 1.94591 | 1.79176 | 22.548 | 9.11637 | 1.39304 | 2.69861 | 4.2535 | 4.7365 |
| 0 | 0 | 6.27099 | 6.06379 | 31.5236 | 51.7571 | 0.19835 | 1.51981 | 0.0544 | 0 |
| 0 | 0 | 6.73221 | 6.41346 | 27.6654 | 51.0841 | 0.10471 | 1.51399 | 0.0544 | 0.1036 |
| 0 | 0 | 5.85507 | 5.9108 | 31.9744 | 48.2631 | 0.12187 | 1.45525 | 0.0544 | 0.2972 |
| 2.6 | 2.6 | 3.7612 | 4.06044 | 33.6357 | 12.9906 | 0.59213 | 1.75088 | 2.4243 | 2.8092 |
| 4.9 | 4.9 | 2.70805 | 2.83321 | 21.5614 | 6.13763 | 0.88074 | 2.43593 | 4.0354 | 4.391 |
| 4.9 | 4.9 | 3.29584 | 3.66356 | 23.2988 | 4.3573 | 0.79479 | 2.59997 | 3.6337 | 3.8686 |

| | | | | | | | | | |
|---|---|---|---|---|---|---|---|---|---|
| 5 | 5 | 1.09861 | 2.94444 | 16.3205 | 2.2575 | 0.65095 | 2.13657 | 4.8535 | 4.988 |
| 0 | 0 | 7.79647 | 7.32185 | 26.9609 | 48.9638 | 0.13146 | 1.52323 | 0.0259 | 0.2904 |
| 0 | 0 | 6.98749 | 6.91274 | 28.5458 | 39.0223 | 0.24219 | 1.72955 | 0.1018 | 0.363 |
| 2 | 2 | 2.83321 | 4.09434 | 37.1239 | 5.1006 | 0.73751 | 2.33853 | 2.436 | 2.5925 |
| 5 | 5 | 1.38629 | 2.56495 | 16.7313 | 1.7308 | 0.66626 | 2.59783 | 4.912 | 4.968 |
| 0 | 0 | 7.87017 | 6.83518 | 25.4424 | 57.6933 | 0.02356 | 1.00787 | 0.0258 | 0.0518 |
| 0 | 0 | 6.62407 | 6.49979 | 27.4077 | 53.9195 | 0.03182 | 0.82066 | 0.0544 | 0.207 |
| 0 | 0 | 5.04343 | 5.77144 | 32.2345 | 51.2795 | 0.33715 | 0.77864 | 0.1658 | 0.1008 |
| 2 | 2 | 2.48491 | 3.52636 | 31.4351 | 6.27597 | 0.62943 | 1.58415 | 2.6043 | 2.9529 |
| 2 | 2 | 2.99573 | 3.97029 | 40.3991 | 8.38297 | 0.66032 | 1.63608 | 2.4247 | 2.8525 |
| 2 | 2 | 2.70805 | 3.52636 | 33.081 | 9.8098 | 0.57956 | 1.58299 | 2.3737 | 2.6309 |
| 2 | 2 | 2.89037 | 2.99573 | 33.88 | 9.81403 | 0.37916 | 1.44739 | 2.4121 | 2.5678 |
| 5 | 5 | 2.07944 | 2.48491 | 19.488 | 5.0015 | 0.66045 | 1.8141 | 4.7135 | 4.811 |

ariable is

| Predicted_Dose_q5 | Predicted_Dose_q10 | Predicted_Dose_q25 | Predicted_Dose_q50 | Predicted_Dose_q75 | Predicted_Dose_q90 | Predicted_Dose_q95 | Predicted_Interaction_q5 | Predicted_Interaction_q10 | Predicted_Interaction_q25 |
|---|---|---|---|---|---|---|---|---|---|
| 0 | 0 | 0 | 0 | 0 | 4.5 | 5.18 | 0 | 0 | 0 |
| 0 | 0 | 0 | 3.2 | 5.16 | 5.7 | 5.7 | 0 | 0 | 0 |
| 2 | 2 | 2 | 2.2 | 2.825 | 4.9 | 5 | 2 | 2 | 2 |
| 0 | 0 | 0 | 0 | 4.925 | 5.7 | 5.7 | 0 | 0 | 0 |
| 2.2 | 2.2 | 4.4 | 4.5 | 5 | 5.25 | 5.25 | 2.2 | 2.2 | 2.2 |
| 0 | 0 | 0 | 0 | 4.9 | 5.18 | 5.51 | 0 | 0 | 0 |
| 0 | 2.2 | 3.2 | 4.5 | 5.25 | 5.7 | 5.7 | 0 | 1.6 | 2 |
| 0 | 0 | 0 | 5.16 | 5.18 | 5.18 | 5.1835 | 0 | 0 | 0 |
| 0 | 0 | 2.6 | 4.5 | 5.16 | 5.5 | 5.7 | 0 | 0 | 1.6 |
| 2 | 2.2 | 3.2 | 4.5 | 5.25 | 5.5 | 5.7 | 0 | 1.6 | 2.2 |
| 0 | 0 | 0 | 0 | 2.8 | 4.928 | 5.7 | 0 | 0 | 0 |
| 0 | 0 | 0 | 0 | 2.6 | 5.18 | 5.7 | 0 | 0 | 0 |
| 2 | 2.2 | 3.2 | 3.2 | 4.625 | 5.7 | 5.7 | 1.6 | 1.6 | 1.6 |
| 0 | 2 | 2.2 | 3.2 | 4.9 | 5.7 | 5.7 | 1.33 | 1.6 | 1.6 |
| 0 | 0 | 0 | 2 | 4.5 | 5.18 | 5.7 | 0 | 0 | 0 |
| 0 | 0 | 0 | 2.59 | 4.5 | 5.7 | 5.7 | 0 | 0 | 0 |
| 0 | 0 | 0 | 0 | 0 | 0 | 0 | 0 | 0 | 0 |
| 0 | 0 | 0 | 0 | 0 | 0 | 0 | 0 | 0 | 0 |
| 0 | 0 | 0 | 0 | 0 | 0 | 0 | 0 | 0 | 0 |
| 0 | 0 | 0 | 0 | 0 | 2.59 | 2.705 | 0 | 0 | 0 |
| 0 | 0 | 0 | 0 | 2.59 | 3.2 | 4.9 | 0 | 0 | 0 |
| 2 | 2 | 2.2 | 3.2 | 4.9 | 4.9 | 5.2725 | 1.4 | 1.6 | 2 |
| 0 | 0 | 0 | 0 | 0 | 3.37 | 5.7 | 0 | 0 | 0 |
| 0 | 0 | 0 | 0 | 0 | 0 | 0 | 0 | 0 | 0 |
| 2 | 2.18 | 2.2 | 2.2 | 2.7 | 3.2 | 4.5 | 1.6 | 1.6 | 2 |
| 2.2 | 3.9 | 4.5 | 4.9 | 5 | 5.25 | 5.25 | 2.2 | 2.2 | 4.9 |
| 0 | 0 | 0 | 0 | 5.16 | 5.18 | 5.18 | 0 | 0 | 0 |
| 2 | 2 | 2.6 | 2.8 | 4.9 | 5.275 | 5.51 | 1.6 | 1.96 | 2.2 |
| 2 | 2 | 2 | 2.6 | 4.5 | 5.16 | 5.1835 | 2 | 2 | 2 |
| 2 | 2.2 | 4.4 | 5 | 5 | 5.5 | 5.5 | 2 | 2 | 2.2 |
| 4.4 | 4.86 | 5 | 5 | 5 | 5.25 | 5.25 | 2.2 | 2.2 | 4.9 |
| 2.2 | 2.2 | 3.9 | 4.9 | 5 | 5.5 | 5.5 | 2 | 2.2 | 2.25 |
| 0 | 0 | 0 | 0 | 0 | 0 | 0 | 0 | 0 | 0 |
| 0 | 0 | 0 | 0 | 0 | 0 | 0 | 0 | 0 | 0 |
| 0 | 0 | 0 | 0 | 0 | 0 | 0 | 0 | 0 | 0 |
| 2 | 2 | 2.6 | 2.6 | 2.7 | 4.9 | 4.905 | 2 | 2.2 | 2.59 |
| 2 | 2.2 | 3.9 | 5 | 5.25 | 5.5 | 5.5 | 2 | 2 | 2.2 |
| 2 | 2 | 2.2 | 3.9 | 5 | 5.25 | 5.5 | 1.6 | 2 | 2 |

| | | | | | | | | | |
|---|---|---|---|---|---|---|---|---|---|
| 2.2 | 4.4 | 5 | 5 | 5 | 5.25 | 5.25 | 2.19 | 2.2 | 4.975 |
| 0 | 0 | 0 | 0 | 0 | 0 | 0 | 0 | 0 | 0 |
| 0 | 0 | 0 | 0 | 0 | 0 | 0 | 0 | 0 | 0 |
| 2 | 2 | 2 | 2 | 2.6 | 3.27 | 5.2625 | 2 | 2 | 2 |
| 3.9 | 4.9 | 5 | 5 | 5 | 5.25 | 5.25 | 2.2 | 4.9 | 5 |
| 0 | 0 | 0 | 0 | 0 | 0 | 0 | 0 | 0 | 0 |
| 0 | 0 | 0 | 0 | 0 | 0 | 0 | 0 | 0 | 0 |
| 0 | 0 | 0 | 0 | 0 | 0 | 2.6005 | 0 | 0 | 0 |
| 2 | 2 | 2 | 2.4 | 4.6 | 5.187 | 5.25 | 2 | 2 | 2 |
| 2 | 2 | 2 | 2.6 | 2.7 | 5.025 | 5.7 | 1.98 | 2 | 2 |
| 2 | 2 | 2.2 | 2.2 | 2.7 | 5.016 | 5.25 | 2 | 2 | 2.2 |
| 2 | 2 | 2 | 2.395 | 2.7 | 5 | 5.25 | 2 | 2 | 2 |
| 2.2 | 2.56 | 4.4 | 5 | 5 | 5.25 | 5.5 | 2 | 2.2 | 3.9 |

| Predicted_Interaction_q50 | Predicted_Interaction_q75 | Predicted_Interaction_q90 | Predicted_Interaction_q95 | Actual_Dose_minus_Interaction | Predicted_Dose_minus_Interaction |
|---|---|---|---|---|---|
| 0 | 0 | 2.25 | 2.59 | 0 | 0.2396 |
| 2.1 | 2.6 | 2.85 | 2.85 | 1.2 | 1.3021 |
| 2.2 | 2.2 | 2.7 | 4.905 | 1.2 | 0.2044 |
| 0 | 2.58 | 2.85 | 2.85 | 1.2 | 1.1291 |
| 2.25 | 2.25 | 5 | 5.25 | 2.7 | 1.4336 |
| 0 | 2.59 | 2.625 | 2.85 | 1.295 | 1.076 |
| 2.35 | 2.7375 | 2.85 | 5 | 1.305 | 1.9439 |
| 2.58 | 2.59 | 2.59 | 2.603 | 1.33 | 1.6739 |
| 2.25 | 2.6 | 2.85 | 3.95 | 2.59 | 1.7812 |
| 2.25 | 2.85 | 5 | 5.25 | 2.605 | 1.8268 |
| 0 | 1.6 | 2.59 | 2.85 | 0 | 0.7488 |
| 0 | 2.25 | 2.6 | 2.85 | 0 | 0.4563 |
| 2 | 2.25 | 2.85 | 2.85 | 1.55 | 1.5771 |
| 2.2 | 2.7375 | 2.85 | 2.85 | 2.8 | 1.4035 |
| 2.25 | 2.59 | 2.715 | 2.85 | 0 | 1.0332 |
| 2.58 | 2.59 | 2.85 | 2.85 | 0 | 1.0585 |
| 0 | 0 | 0 | 0 | 0 | 0.0233 |
| 0 | 0 | 0 | 0 | 0 | 0.0171 |
| 0 | 0 | 0 | 0 | 0 | 0.0611 |
| 0 | 0 | 1.42 | 2.59 | 1.4 | 0.5785 |
| 0 | 1.4 | 2.45 | 2.59 | 1.35 | 0.7971 |
| 2.25 | 2.6 | 2.715 | 2.85 | 2.55 | 1.2103 |
| 0 | 0 | 2.59 | 2.85 | 2.5 | 0.4921 |
| 0 | 0 | 0 | 0 | 0 | -0.052 |
| 2.2 | 2.7 | 2.7 | 2.7075 | 0 | 0.4397 |
| 4.9 | 5 | 5.25 | 5.25 | 0 | 0.1125 |
| 0 | 0 | 2.59 | 2.59 | 0 | 0.3785 |
| 2.45 | 2.7 | 4 | 5.25 | 0 | 0.7046 |
| 2.2 | 2.7 | 4.91 | 5.25 | 0 | 0.4518 |
| 3.9 | 5 | 5.5 | 5.5 | 0 | 0.677 |
| 5 | 5 | 5.25 | 5.25 | 0 | 0.1205 |
| 4.9 | 5.3125 | 5.5 | 5.5 | 0 | 0.483 |
| 0 | 0 | 0 | 0 | 0 | -0.0544 |
| 0 | 0 | 0 | 0 | 0 | 0.0492 |
| 0 | 0 | 0 | 0 | 0 | 0.2428 |
| 2.6 | 2.7 | 2.7 | 2.9575 | 0 | 0.3849 |
| 3.9 | 5 | 5.5 | 5.5 | 0 | 0.3556 |
| 3.9 | 5 | 5.25 | 5.2625 | 0 | 0.2349 |

| | | | | | |
|---|---|---|---|---|---|
| 5 | 5 | 5.25 | 5.25 | 0 | 0.1345 |
| 0 | 0 | 0 | 0 | 0 | 0.2645 |
| 0 | 0 | 0 | 0.07 | 0 | 0.2612 |
| 2.1 | 2.4875 | 2.85 | 4.905 | 0 | 0.1565 |
| 5 | 5 | 5.25 | 5.25 | 0 | 0.056 |
| 0 | 0 | 0 | 0 | 0 | 0.026 |
| 0 | 0 | 0 | 0 | 0 | 0.1526 |
| 0 | 0 | 0 | 0 | 0 | -0.065 |
| 2.2 | 3.1125 | 5.25 | 5.25 | 0 | 0.3486 |
| 2.45 | 2.6 | 2.85 | 5 | 0 | 0.4278 |
| 2.2 | 2.625 | 5.025 | 5.25 | 0 | 0.2572 |
| 2.2 | 2.625 | 4 | 5.25 | 0 | 0.1557 |
| 5 | 5 | 5.25 | 5.5 | 0 | 0.0975 |
