# Supplementary material for: Machine learning approach for quantitative biodosimetry of partial-body or total-body radiation exposures by combining radiation-responsive biomarkers
Source: Sci Rep. 2023 Jan 18;13:949. doi: 10.1038/s41598-023-28130-0 (PMC9849198; doi:10.1038/s41598-023-28130-0)
Supplement: Supplementary file 3 — Supplementary Information 3. [file 41598_2023_28130_MOESM3_ESM.pdf]

Supplementary table S3. Comparison of the observed data in the testing data set with RF model predictions.											
Interaction	Dose	ln B cells	ln T cells	Percent B cells	Percent T cells	DDB2	FDXR	Exposed indicator	RF predicted exposure probabilit	RF predicted exposed indicator
0.00	0.00	4.74	5.06	54.30	26.83	-0.53	1.99	No	0.18	No
1.20	2.40	3.00	3.95	49.55	30.74	-0.47	2.34	Yes	0.71	Yes
1.20	2.40	2.48	2.83	12.33	32.72	0.62	2.92	Yes	0.96	Yes
1.20	2.40	3.47	4.03	51.52	24.25	-0.52	1.97	Yes	0.51	Yes
2.70	5.40	2.30	2.83	16.25	18.16	0.27	3.15	Yes	0.96	Yes
1.30	2.59	4.34	4.56	52.35	21.40	0.97	1.17	Yes	0.51	Yes
1.31	2.61	2.77	3.66	37.39	22.64	-0.11	1.47	Yes	0.51	Yes
1.33	2.66	4.32	4.48	43.34	28.40	0.04	1.10	Yes	0.51	Yes
2.59	5.18	2.89	3.71	36.52	24.83	-0.34	1.12	Yes	0.80	Yes
2.61	5.21	2.64	3.30	31.80	23.31	0.15	1.49	Yes	0.80	Yes
0.00	0.00	4.74	4.92	42.39	38.46	0.95	1.99	No	0.51	Yes
0.00	0.00	4.17	4.88	45.17	34.32	0.63	1.79	No	0.18	No
1.55	3.10	2.83	3.14	38.01	40.43	1.03	2.24	Yes	0.71	Yes
2.80	5.60	2.77	3.37	36.28	34.44	0.84	1.91	Yes	0.80	Yes
0.00	0.00	3.99	4.61	46.53	32.11	0.75	1.93	No	0.51	Yes
0.00	0.00	3.61	4.41	51.62	31.47	0.40	1.76	No	0.18	No
0.00	0.00	6.68	5.88	53.67	29.67	0.15	1.59	No	0.13	No
0.00	0.00	6.29	5.95	49.84	34.46	0.48	1.52	No	0.13	No
0.00	0.00	6.06	5.63	47.69	31.62	0.27	1.64	No	0.13	No
1.40	2.80	4.88	5.22	39.11	34.59	0.44	1.74	Yes	0.51	Yes
1.35	2.70	4.71	5.11	34.77	37.09	0.37	1.87	Yes	0.47	No
2.55	5.10	3.61	3.33	20.96	30.49	0.51	2.04	Yes	0.96	Yes
2.50	5.00	4.77	4.86	43.46	38.00	0.31	1.53	Yes	0.80	Yes
0.00	0.00	5.92	5.94	57.35	26.17	-0.68	1.81	No	0.00	No
2.70	2.70	3.50	3.64	9.70	39.04	0.12	3.10	Yes	0.96	Yes
4.30	4.30	1.79	2.20	3.19	21.43	0.25	3.29	Yes	1.00	Yes
0.00	0.00	4.39	4.79	51.50	24.64	-0.45	0.93	No	0.51	Yes
2.55	2.55	3.93	2.56	18.77	33.79	0.13	1.44	Yes	1.00	Yes
2.57	2.57	3.30	2.83	9.51	32.18	-0.14	1.19	Yes	1.00	Yes
5.21	5.21	2.48	3.09	9.57	17.28	1.10	1.80	Yes	1.00	Yes
5.24	5.24	1.39	2.08	3.02	15.21	0.38	1.80	Yes	1.00	Yes
5.50	5.50	1.95	1.79	9.12	22.55	1.39	2.70	Yes	1.00	Yes
0.00	0.00	6.27	6.06	51.76	31.52	0.20	1.52	No	0.13	No
0.00	0.00	6.73	6.41	51.08	27.67	0.10	1.51	No	0.13	No
0.00	0.00	5.86	5.91	48.26	31.97	0.12	1.46	No	0.13	No
2.60	2.60	3.76	4.06	12.99	33.64	0.59	1.75	Yes	1.00	Yes
4.90	4.90	2.71	2.83	6.14	21.56	0.88	2.44	Yes	1.00	Yes
4.90	4.90	3.30	3.66	4.36	23.30	0.79	2.60	Yes	1.00	Yes
5.00	5.00	1.10	2.94	2.26	16.32	0.65	2.14	Yes	0.96	Yes

0.00	0.00	7.80	7.32	48.96	26.96	0.13	1.52	No	0.13	No
0.00	0.00	6.99	6.91	39.02	28.55	0.24	1.73	No	0.13	No
2.00	2.00	2.83	4.09	5.10	37.12	0.74	2.34	Yes	1.00	Yes
5.00	5.00	1.39	2.56	1.73	16.73	0.67	2.60	Yes	1.00	Yes
0.00	0.00	7.87	6.84	57.69	25.44	0.02	1.01	No	0.00	No
0.00	0.00	6.62	6.50	53.92	27.41	0.03	0.82	No	0.13	No
0.00	0.00	5.04	5.77	51.28	32.23	0.34	0.78	No	0.18	No
2.00	2.00	2.48	3.53	6.28	31.44	0.63	1.58	Yes	1.00	Yes
2.00	2.00	3.00	3.97	8.38	40.40	0.66	1.64	Yes	1.00	Yes
2.00	2.00	2.71	3.53	9.81	33.08	0.58	1.58	Yes	1.00	Yes
2.00	2.00	2.89	3.00	9.81	33.88	0.38	1.45	Yes	1.00	Yes
5.00	5.00	2.08	2.48	5.00	19.49	0.66	1.81	Yes	1.00	Yes
